# Enhancing Slurry Stability and Surface Flatness of Silicon Wafers through Organic Amine-Catalyzed Synthesis Silica Sol

**DOI:** 10.3390/nano14161371

**Published:** 2024-08-22

**Authors:** Yi Xing, Weilei Wang, Weili Liu, Zhitang Song

**Affiliations:** 1 State Key Laboratory of Materials for Integrated Circuits, Shanghai Institute of Microsystem and Information Technology, Chinese Academy of Sciences, Changning, Shanghai 200050, China; xingy@mail.sim.ac.cn (Y.X.); ztsong@mail.sim.ac.cn (Z.S.); 2 University of Chinese Academy of Sciences, Shijingshan, Beijing 100049, China; 3 Zhejiang Xinchuangna Electronic Technology Co., Ltd., Haining 314406, China; awelly@mail.sim.ac.cn

**Keywords:** monocrystalline silicon wafer, chemical mechanical polishing, organic amine, silanol groups, silica nanoparticles

## Abstract

The stability of slurries used for chemical mechanical polishing (CMP) is a crucial concern in industrial chip production, influencing both the quality and cost-effectiveness of polishing fluids. In silicon wafer polishing, the conventional use of commercial neutral silica sol combined with organic bases often leads to slurry instability. To address this issue, this study proposes organic amines—specifically ethanolamine (MEA), ethylenediamine (EDA), and tetramethylammonium hydroxide (TMAOH)—as catalysts for synthesizing alkaline silica sol tailored for silicon wafer polishing fluids. Sol–gel experiments and zeta potential measurements demonstrate the efficacy of this approach in enhancing the stability of silica sol. The quantitative analysis of surface hydroxyl groups reveals a direct correlation between enhanced stability and increased hydroxyl content. The application of the alkaline silica sol in silicon wafer polishing fluids improves polishing rates and enhances surface flatness according to atomic force microscopy (AFM). In addition, electrochemical experiments validate the capability of this polishing solution to mitigate corrosion on silicon wafer surfaces. These findings hold significant implications for the advancement of chemical mechanical polishing techniques in the field of integrated circuit fabrication.

## 1. Introduction

With the rapid development of artificial intelligence, cloud computing, and the Internet of Things, there is an increasing demand for high-performance and reliable semiconductor devices [1]. Silicon wafers, as a first-generation substrate material, exhibit excellent thermal and crystalline structural stability and remain extensively employed in fabricating high-power devices within integrated circuits (ICs). In silicon wafer production, the grinding and polishing stages are critical. They effectively remove the damage layer caused by the cutting process, resulting in a smooth surface that enhances subsequent device manufacturing and improves the performance of the final product [2]. Through-silicon via (TSV) is an advanced three-dimensional interconnect technology that greatly improves electrical performance through vertical interconnect modules [3]. It has been widely used in Micro-Electro-Mechanical Systems (MEMSs), memory, image sensor power amplifiers, and other fields. In order to vertically bond more chips using TSV technology, the silicon wafers are required to be as thin as possible [4,5].

Colloidal silica nanoparticles emerge as the preferred abrasive for silicon wafer chemical mechanical polishing (CMP) due to the properties of moderate hardness, excellent dispersibility, and stability [6]. The sol–gel method is the most common method to synthesize the ultra-high-purity silica sol without metal ion impurities. Stöber et al. [7] established the synthesis route to produce silica nanoparticles with variable particle sizes between 5 and 2000 nm by changing the reaction conditions in 1968. Since then, many studies based on Stöber silica particles have investigated the effect of reaction conditions on the formation and growth mechanism of silica particles [8,9,10,11]. Ammonia (NH_4_OH) was the most common catalyst to promote the hydrolysis and condensation of TEOS to fabricate silica particles [12,13]. Organic bases have emerged as promising substitutes for NH_4_OH in silica sol synthesis due to their comparable catalytic activity. Dong et al. [14] introduced tetrabutylammonium hydroxide (TBAOH) as a co-catalyst of NH_4_OH to synthesize fractal silica particles with tunable dimensions by changing the concentration of TBAOH. Manuel and co-workers [15] investigated ethanolamine (MEA) as a catalyst to synthesize silica particles with a narrow distribution at high reaction temperatures, and they studied the effect of ethylenediamine concentration on reaction kinetics at different temperatures. In addition, basic amino acids such as arginine and lysine have also been effectively used as catalysts in modified Stöber synthesis [16,17,18].

Numerous experiments have demonstrated that the integration of organic amines with silica abrasives can enhance the polishing rate of silicon wafers effectively. Bae et al. [2] demonstrated that using organic amines as accelerators in silicon wafer polishing significantly increased the polishing rate and decreased surface roughness compared to using inorganic bases. This enhancement was credited to the formation of Si-N bonds between the amine groups and the silicon surface. This interaction reduced the contact angle between the polishing slurry and the silicon wafer surface, thereby promoting the adsorption of the CMP slurry onto the silicon wafer surface. Furthermore, organic amines not only eliminated the risk of metal ion contamination but also served as chelating agents to remove metal ion impurities. This addressed concerns such as equipment penetration, increased leakage current, reduced chip reliability, and equipment inefficiency. Although previous studies have shown that incorporating organic amines into silica sol as silicon wafer polishing slurries achieved good polishing rates and surface flatness, according to our previous research, introducing organic amines into neutral silica sol within pH 8.00–11.00 can lead to the instability of the silica sol, resulting in gelation in a short period of time [19].

Therefore, in this study, ethanolamine (MEA), ethylenediamine (EDA), and tetramethylammonium hydroxide (TMAOH) were employed as alternative catalysts to NH_4_OH in the synthesis of silica. The resulting alkaline silica sol was subsequently employed in the chemical mechanical polishing process of single-crystal silicon wafers. The stability of the silica sol was evaluated through sol–gel experiments and zeta potential measurements. The quantitative determination of the silica hydroxyl content facilitated the analysis of stability variations under diverse synthesis conditions. Moreover, a comprehensive investigation of silanol groups, silicon network cross-linking degree, internal hydroxyl groups, and porosity properties was conducted using thermogravimetric analysis (TGA), ^29^Si solid-state NMR spectroscopy, and nitrogen adsorption tests. Subsequently, silica sol synthesized at pH 10.50 was applied for silicon wafer polishing, with surface roughness assessed using atomic force microscopy (AFM) and the corrosion impact of the polishing solution on silicon wafers evaluated via electrochemical experiments.

## 2. Materials and Methods

### 2.1. Experimental Details

#### 2.1.1. Synthesis of Silica Nanoparticles Using Organic Amines as Catalysts

The ethanol solvent (Jiangsu Qiangsheng Chemical Co. Ltd., Suzhou, China, ≥99.7%) was divided into two equal volumes. One portion was used to prepare stock solution A by mixing with tetraethyl orthosilicate (TEOS, Sinopharm Chemical Reagent Co., Ltd., Shanghai, China, ≥28.4%) while the other portion was mixed with organic amine and water as stock solution B. The organic amines used were ethanolamine (MEA, Macklin Biochemical Co., Ltd., Shanghai, China, >99%), ethylenediamine (EDA, Yonghua Chemical Co., Ltd., Suzhou, China, ≥99%), and tetramethylammonium hydroxide (TMAOH, Aladdin Biochemical Technology Co., Ltd., Shanghai, China, 10%). The pH values of the final synthesized silica sol with each catalyst were controlled to 9.00, 9.50, 10.00, 10.50, and 11.00, respectively. The concentration of TEOS was 0.33 M and the concentration of water was 3 M. Prior to the reaction, both solution A and solution B were preheated to 25 °C. Solution B was then rapidly added to solution A and vigorously shaken. After an 8 h reaction period at 25 °C with magnetic stirring at 600 rpm, the obtained particles were purified using evaporating ethanol, resulting in a silica solid content of 1wt% in the aqueous phase. The alkaline silica sols synthesized using organic bases as the catalysts were used as the experimental groups.

Commercial silicon oxide (FUSO Chemical Co., Ltd., Tokyo, Japan, 19.67 wt%), initially with a pH of 7.67, was adjusted to pH values of 9.00, 9.50, 10.00, 10.50, and 11.00 using TMAOH as a pH adjuster for the control group.

#### 2.1.2. Determination of Hydroxyl Content of Silica Nanoparticles

According to the solid content of the dispersed silica solution in both the control and experimental groups, 2.0000 g of silica was weighed and transferred into a 250 mL beaker. Subsequently, 25.00 mL of ethanol and 75.00 mL of a 20% sodium chloride solution (NaCl, Aladdin Biochemical Technology Co., Ltd., Shanghai, China, 99.5%) were added and mixed thoroughly. Then, the pH of the mixture was adjusted to 4.00 using a 0.1 M hydrochloric acid (HCl, Aladdin Biochemical Technology Co., Ltd., China, 37%) or sodium hydroxide solution (NaOH, Aladdin Biochemical Technology Co., Ltd., China, 97%). Titration proceeded slowly with a 0.1 M NaOH solution until the pH reached 9.00 and was maintained at 9.00 for 20 s to ensure its stability. The formula for calculating the hydroxyl content of silica is given in Equation (1):

(1) $$N=C\cdot V\cdot 10^{-3}$$

wherein *N* represents the number of moles of hydroxyl groups per gram of silica nanoparticles (mol/g), *C* represents the concentration of NaOH standard titration solution (M), and *V* is the volume of NaOH standard titration solution consumed during titration from pH 4.00 to 9.00 (mL).

#### 2.1.3. Sol–Gel Experiment

The 5000 ppm NaCl solution with a concentration of 5% was added to both the control group and the experimental groups, followed by thorough stirring. Subsequently, silica sol was aged in a 45 °C oven to expedite the process. Upon reaching the gel point, significant changes in the rheological properties of the colloidal system were observed, indicative of loss of fluidity, and the sol–gel transition time was recorded.

#### 2.1.4. CMP Experiment

The 8-inch silicon wafers (100) were polished using a JHY-005 polishing pad manufactured by Wuxi NuoQiusheng Electronic Technology Co., Ltd., Wuxi, China, on a 50 GPAW polishing machine from Chuangji Electronic Machinery (Shanghai) Co., Ltd., China. The primary polishing parameters are detailed in Table 1. The polishing slurry consisted of four groups including one control group and three experimental groups, all adjusted to a pH of 10.50.

### 2.2. Characterization Methods

#### 2.2.1. Characterizations of Silica Particles

The zeta potential of colloid silica was measured using a zeta potential analyzer (Zeta Acoustic ZA500, Mass Applied Sciences, Hopedale, MA, USA). Thermogravimetric analysis (TGA) was performed with a Netzsch TG 209 F3 Tarsus instrument under a nitrogen atmosphere. Samples were heated from 30 to 800 °C at a rate of 10 °C /min in a Pt-Rh crucible to assess weight loss and final solid content. The ^29^Si NMR spectra were acquired using an Agilent 600 DD2 spectrometer (Agilent, Texas, USA, magnetic field strength 14.1T) with a Larmor frequency of 199.13 MHz. The experimental parameters included a relaxation delay of 3 s, a cross-polarization (CP) contact time of 2 ms, a 90-degree pulse width of approximately 4 µs, and 1024 scans. Chemical shifts were referenced to tetramethylsilane (TMS) at 0 ppm.

Nitrogen adsorption and desorption at the gas/solid interface enabled the evaluation of a broad spectrum of pore sizes, encompassing both micropores and mesopores using a Micromeritics ASAP 2460 surface analyzer under continuous adsorption conditions at 77 K. Prior to measurement, samples were degassed at 150 °C for 12 h. The specific surface area was determined using the Brunauer–Emmett–Teller (BET) method. Pore size distributions were obtained from the Barrett–Joyner–Halenda (BJH) adsorption branches of the isotherms. The total pore volumes were estimated based on the amounts adsorbed at a relative pressure of 0.995. The micropore area and volume were calculated using the t-Plot method.

#### 2.2.2. Characterizations of Silicon Polishing

The material removal rate was determined by measuring the mass variation in the silicon wafer before and after polishing using an electronic analytical balance with a precision of 0.0001 g, according to Equation (2).

(2) $$MRR=\frac{10^{7}\times ∆m}{\rho \times ∆s\times t}$$

Here, Δ*m* (g) is the mass variation in the silicon wafer before and after polishing, *t* (min) is the polishing time, ρ (g/cm^3^) is the silicon wafer density, Δ*s* (cm^2^) is the surface area of the Si wafer, and *MRR* (nm/min) is the material removal rate.

The surface topography and surface roughness (Ra) were evaluated via atomic force microscopy (AFM, XE-100, Park Systems, Suwon, Republic of Korea) with a scan area of 5 × 5 µm^2^. Both MRR and Ra values were averaged from three independent polishing tests.

The potentiodynamic polarization experiment was conducted using an Autolab Electrochemical Workstation (manufactured by Metrohm Ltd., Herisau, Switzerland). The experimental setup utilized a three-electrode etching cell, comprising a 10 × 10 cm^2^ platinum counter electrode, an Ag/AgCl reference electrode, and the 2-inch silicon wafer as the working electrode. The scan rate was set at 0.5 mV/s, with a potential range of −0.6 to 0.6 V.

## 3. Results and Discussion

### 3.1. Stability Analysis of Organic Amine-Catalyzed Silica

**Figure 1:**
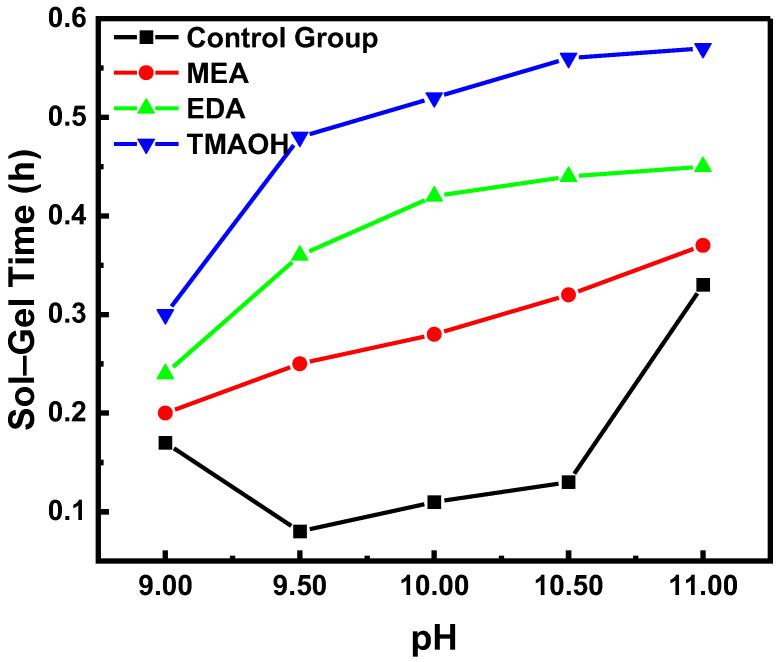
The variation in sol–gel time over pH in the control and experimental groups.

The sol–gel time is a common method used to assess the stability of colloidal dispersions. Generally, longer sol–gel times indicated better colloidal stability. Figure 1 shows that the sol–gel times of the experimental groups at different pH levels were significantly longer compared to the control group. This suggested that the stability of silica sol synthesized directly with organic amines as catalysts was superior to that of neutral silica sol with added alkali. Within the pH range of 9.00–11.00, the stability of the control group initially decreased and then increased as pH increased, a trend explained by our previous experiments using the non-DLVO theory [19]. In contrast, the stability of silica sol in the experimental groups continued to increase. In addition, at equivalent pH levels, the stability of silica synthesized with different organic amines as catalysts varied notably and decreased in the following order: TMAOH exhibited the highest stability, followed by EDA and then MEA.

**Figure 2:**
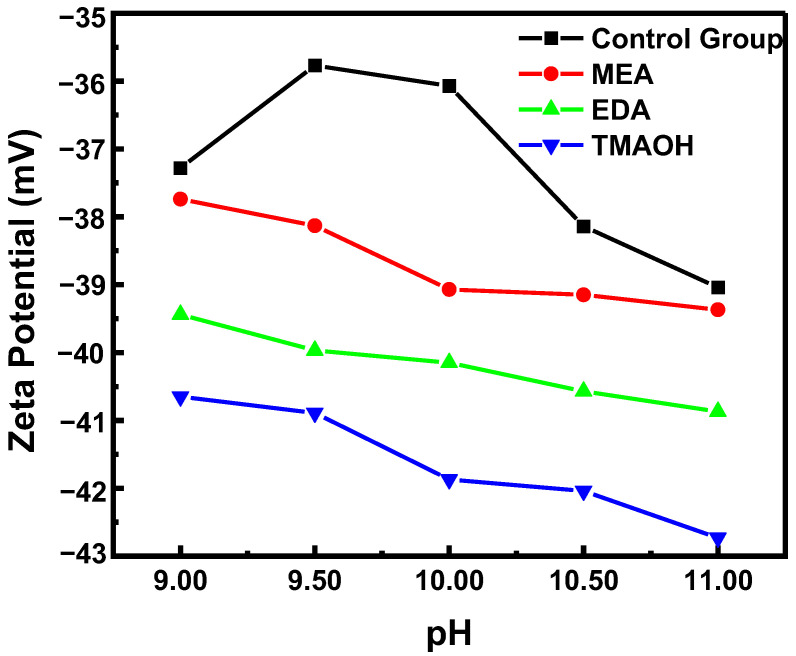
The variation in zeta potential over pH in the control and experimental groups.

The change in zeta potential also reflected the stability of silica sol, with a higher absolute zeta potential indicating better colloidal stability. In the control group, the absolute zeta potential initially decreased and then increased with increasing pH, as shown in Figure 2. In contrast, the absolute zeta potential of silica sol in experimental groups showed a continuous increase, consistent with the results from sol–gel experiments. Furthermore, at the same pH, the absolute zeta potential values of silica sol synthesized using three different organic bases ranked highest to lowest as follows: TMAOH, EDA, and MEA.

### 3.2. Analysis of Silanol Groups of Silica Particles

#### 3.2.1. Quantification Testing of Hydroxyl Content

**Figure 3:**
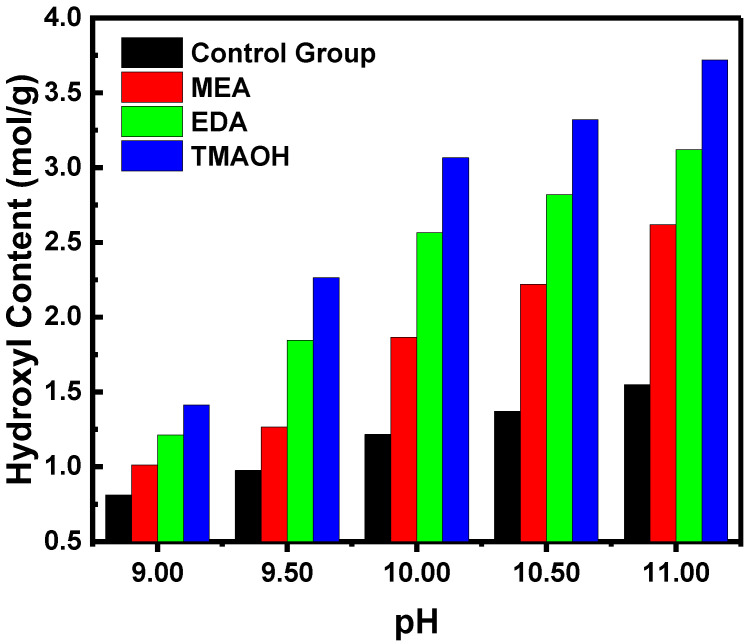
The variation in hydroxyl content over pH in the control and experimental groups.

The reasons for enhanced stability were further elucidated by quantitatively analyzing the hydroxyl content of silica. Silica synthesized using organic amines as catalysts exhibited significantly higher hydroxyl content compared to the control group (Figure 3). Meanwhile, increasing concentrations of organic amines corresponded to higher hydroxyl content. Additionally, catalysts with equal pH of MEA, EDA, and TMAOH showed notable variations in hydroxyl concentration. Silica synthesized with TMAOH as the catalyst demonstrated the highest hydroxyl concentration, followed sequentially by EDA and MEA. These results underscored that increased hydroxyl content substantially enhanced the stability of silica sol, consistent with prior sol–gel experiments and zeta potential data.

#### 3.2.2. TGA

**Figure 4:**
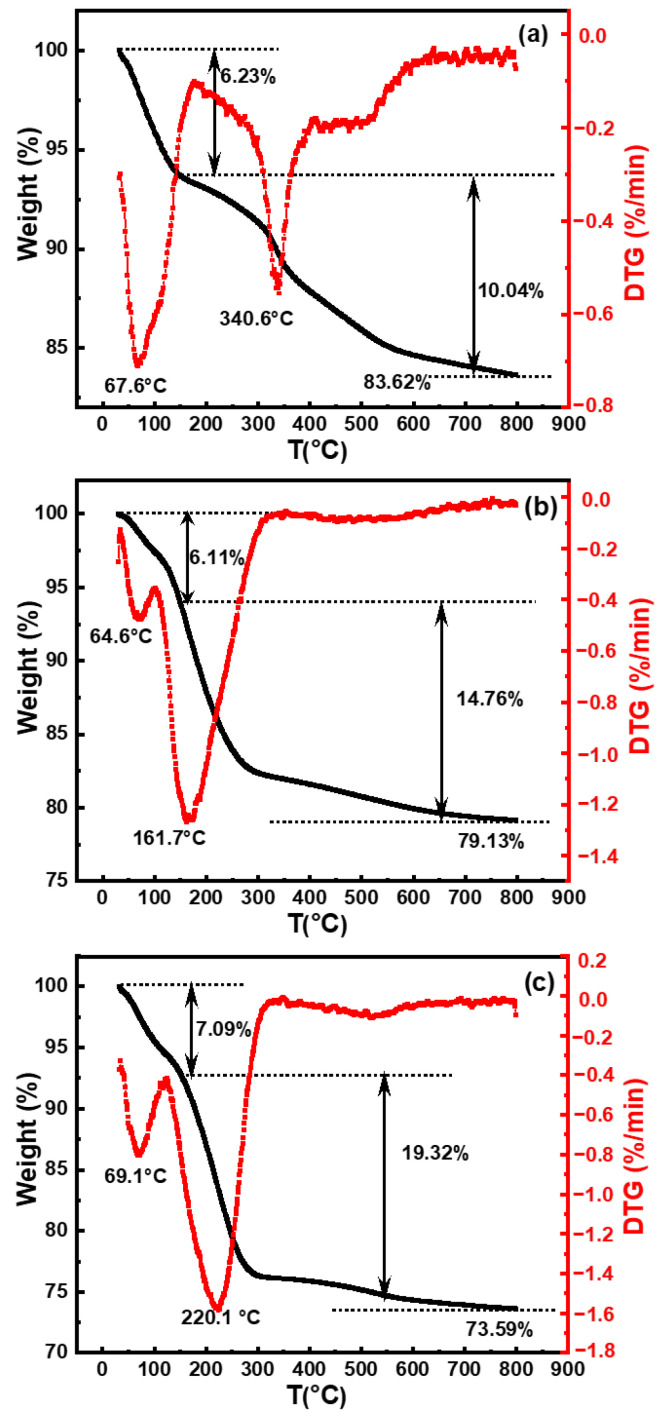
Thermogravimetric analysis (TGA, black line) and derivative thermogravimetry (DTG, red line) curves of SiO_2_ synthesized with different organic amine catalysts at pH = 10.50: (**a**) MEA, (**b**) EDA, and (**c**) TMAOH.

The pH commonly used for silicon wafer polishing solutions was 10.50 [20]. Previous analyses have indicated that the stability of silica sol increased within the pH range of 9.00–11.00. To compare differences in synthesized silica under various catalysts, samples selected for TGA consisted of silica particles synthesized with organic amines (MEA, EDA, and TMAOH) at pH 10.50, as depicted in Figure 4.

It was observed that silica particles from these three systems exhibited two stages of weight loss at temperatures ranging from 30 to 150 °C and 150 to 800 °C, respectively. The initial weight loss primarily originated from the desorption of physical adsorption species and residual organic components [21], which could be eliminated through heating-induced evaporation. Silica particles in these three systems exhibited similar initial weight losses of ca. 6.32%, 6.11%, and 7.09%, respectively. The second weight loss stage, occurring between 150 °C and 800 °C, was attributed to the removal of silanol groups [10]. The hydroxyl content of silica synthesized using TMAOH as catalyst was 19.32%, which was significantly higher than the hydroxyl contents of silica synthesized with MEA and EDA as catalysts, which were 10.04% and 14.76%, respectively. Based on the TGA results, the final contents of silica were determined to be approximately ca. 83.62 wt.%, 79.13 wt.%, and 73.59 wt.%, respectively, for MEA, EDA, and TMAOH as catalysts. These findings from TGA further illustrate the differences in surface silicon hydroxyl content of silica synthesized with different organic amines as catalysts at the same pH. Silica synthesized with TMAOH catalysis exhibited a richer surface of silicon hydroxyl groups compared to those prepared with EDA and MEA.

#### 3.2.3. The ^29^Si Solid-State NMR Analysis

**Figure 5:**
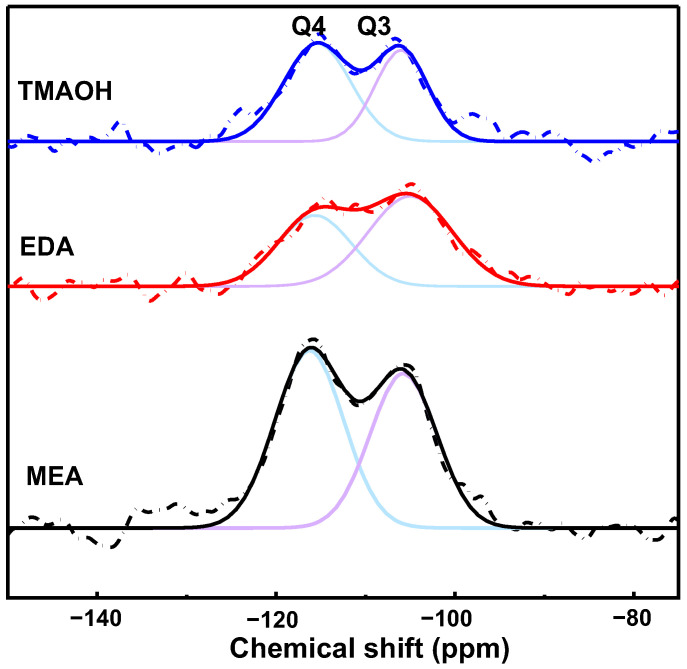
Solid-state ^29^Si NMR spectra of the SiO_2_ synthesized with different organic amine catalysts at pH = 10.50 (Q3, purple line; Q4, blue line).

The ^29^Si solid-state NMR spectra provided quantitative insights into the cross-linking of the silica network structure by examining the ratio of Q4/Q3 [18], as depicted in Figure 5. In these spectra, signals at −102 ppm and −112 ppm were assigned to Q3 and Q4 species [14], which were represented by purple and blue lines, respectively. In NMR terminology, Qn denotes silicon atoms where *n* indicates the number of bridging oxygens bonded to the central silicon. Specifically, Q4 refers to silicon atoms with four Si-O-Si bonds, while Q3 denotes silicon atoms with three Si-O-Si bonds and one Si-OH bond.

The calculated Q4/Q3 ratios were determined as 1.18, 0.69, and 1.35 for silica synthesized using MEA, EDA, and TMAOH as catalysts, respectively. These ratios provided valuable insights into the extent of cross-linking within the silica network. Higher Q4/Q3 ratios generally indicated a higher degree of cross-linking, suggesting that silica synthesized with TMAOH as a catalyst exhibited a more interconnected network compared to those synthesized with MEA and EDA.

#### 3.2.4. Micropore and Internal Hydroxyls Analysis

Legrand et al. [22] noted the dependence of the silanol content on internal silanol groups and the intricate surface structure characterized by a highly heterogeneous distribution of hydroxyls, influenced by the presence of polysilicic acid. Silicon dioxide features hydroxyl groups not only on its surface but also internally. Hence, a thorough analysis of both surface and internal hydroxyl groups in silica was conducted using N_2_ adsorption and pore size distribution analysis.

Internal hydroxyl groups engage in hydrogen bonding with water molecules. The removal of these hydrogen-bonded water molecules contributes to the formation of small micropores within the silica structure [23]. As shown in Table 2, silica synthesized with TMAOH as a catalyst exhibited significantly larger S_micro_/S_BET_ (58.39%) and V_micro_/V_t_ (2.41%) ratios compared to MEA (S_micro_/S_BET_ = 12.48%, V_micro_/V_t_ = 0.93%) and EDA (S_micro_/S_BET_ = 7.48%, V_micro_/V_t_ = 0.79%), confirming TMAOH-catalyzed synthesis enriched the silica with abundant internal hydroxyl groups.

#### 3.2.5. The Adsorption between Organic Amine and Silanol Groups

**Figure 6:**
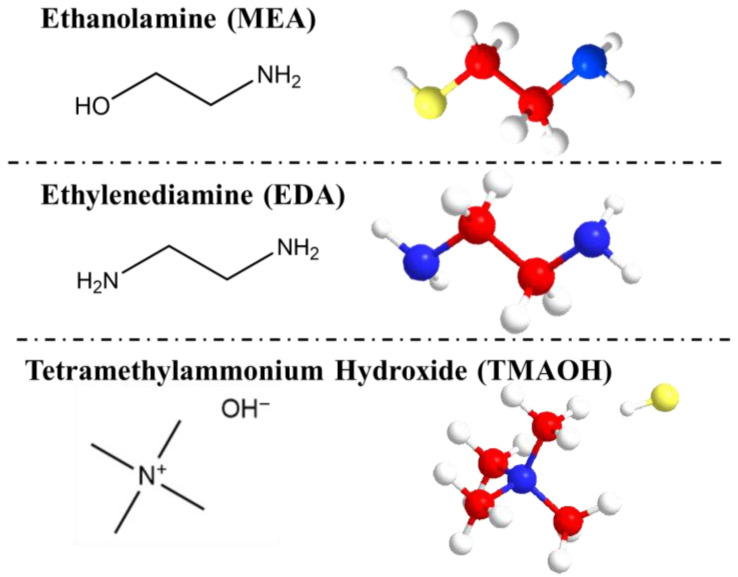
Structural formulas and 3D models of different organic amines (Carbon atom, red; Oxygen atom, yellow; Nitrogen atom, blue; Hydrogen atom, white).

Based on the preceding analysis of silanol groups, a detailed exposition of the mode of action of organic amines on silica was presented. The molecular structural formulas and 3D models of three organic amine molecules are displayed in Figure 6, emphasizing the amino and hydroxyl groups.

**Figure 7:**
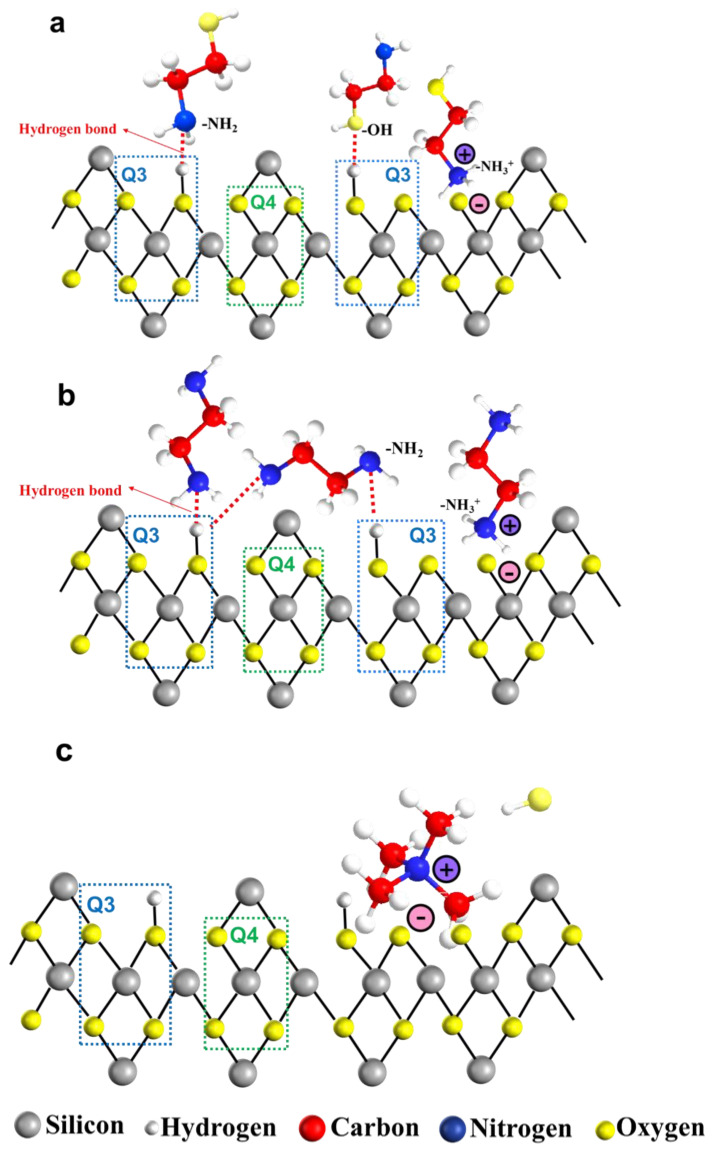
The adsorption between organic amine molecules and silanol groups: (**a**) MEA, (**b**) EDA, and (**c**) TMAOH.

The adsorption of organic amine molecules and ions on the silica surface is depicted in Figure 7. The ionization behavior of organic amines in aqueous environments is represented by Equations (3)–(5). Specifically, amino and hydroxyl groups in MEA can engage in hydrogen bonding with silicon hydroxyl groups present on the silica surface. Under alkaline conditions, surface hydroxyl groups on silica are neutralized by hydroxide ions (-OH), yielding water molecules and thereby generating oxygen ions. Concurrently, the amino groups can ionize in water to form ammonium ions(-NH_3_^+^), which subsequently interact with the oxygen ions (-Si-O-) following the dehydration of the silicon hydroxyl groups (Figure 7a). The adsorption behavior of EDA on silica surfaces resembled that of MEA. The amino groups (-NH_2_) on both sides of EDA molecules can form hydrogen bonds with silicon hydroxyl groups in different arrangements, while the ionized ammonium ions can interact with the negatively charged surface of silicon dioxide via electrostatic forces (Figure 7b) [24].

Notably, according to findings from ^29^Si NMR spectroscopy, surface silicon hydroxyl groups (Si-OH) on silica predominantly existed in two forms, Q3 and Q4. The hydrogen-bonding interactions between organic amine molecules and silicon hydroxyl groups primarily occurred on Q3 sites. This observation suggested that higher Q3 content provided more binding sites conducive to hydrogen bond formation.

In contrast, the presence of TMAOH on silica surfaces differed from MEA and EDA. TMAOH is an ionic compound that can completely ionize into tetramethylammonium ions (TMA^+^) and hydroxide ions (OH^−^) in aqueous solution (Equation (5)). TMA^+^ ions are directly associated with negatively charged silica surfaces through electrostatic attraction without forming hydrogen bonds (Figure 7c). Furthermore, organic amine acted as catalysts to facilitate the hydrolysis of TEOS by supplying OH^ࢤ^ ions (Equation (6)) and the subsequent condensation of hydrolysis products to promote the formation of silicon–oxygen bonds, ultimately yielding silica particles (Equations (7) and (8)).

(3) $$NH_{2}CH_{2}CH_{2}OH+H_{2}O↔NH_{3}^{+}CH_{2}CH_{2}OH+OH^{-}$$

(4) $$\;\;\;\;\;\;\;\;\;\;NH_{2}CH_{2}CH_{2}NH_{2}+2H_{2}O↔NH_{3}^{+}CH_{2}CH_{2}NH_{3}^{+}+2OH^{-}$$

(5) $$\;\;\;\;\;\;\;\;\;\;\;\;\;\;\;\;\;\;\;\;\;\;\;\;\;\;N(CH_{3}{)}_{4}OH\to N(CH_{3}{)}_{4}^{+}+OH^{-}$$

(6) $$Hydrolysis:\;\;\;\;Si(OC_{2}H_{5}{)}_{4}+{nH}_{2}O\overset{organic\;amines}{\to }Si(OH{)}_{n}(OC_{2}H_{5}{)}_{4-n}+{nC}_{2}H_{5}OH$$

(7) $$\;\;\;Condensation:\;\;\;\;Si(OC_{2}H_{5}{)}_{4}+Si(OH{)}_{n}(OC_{2}H_{5}{)}_{4-n}\overset{organic\;amines}{\to }SiO_{2}+C_{2}H_{5}OH$$

(8) $$\;Condensation:\;\;\;\;Si(OH{)}_{4}+Si(OH{)}_{4}\overset{organic\;amines}{\to }SiO_{2}+H_{2}O$$

### 3.3. Application of Silica Sol with pH = 10.50 in Silicon Wafer Polishing

#### 3.3.1. Material Removal Rate and Surface Morphology Analysis

**Figure 8:**
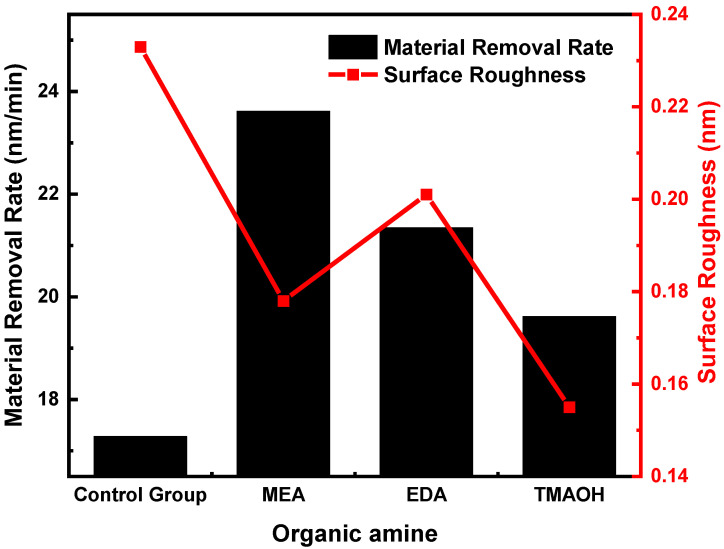
Effect of the silicon polishing slurry from the control group and experimental groups on MRR and Ra at pH = 10.50.

The material removal rate (MRR) and surface roughness (Ra) of silicon wafers using the control group and experimental group polishing slurry with a pH of 10.50 are shown in Figure 8. The analysis of MRR data clearly demonstrated that silica sol in experimental groups achieved significantly higher removal rates in comparison to the control group. A higher hydroxyl content was more favorable for surface protection, which led to a decrease in the polishing rate as the hydroxyl content increased. Therefore, the polishing rate was in the following order: MEA > EDA > TMAOH.

Moreover, the analysis of surface roughness data revealed that the colloidal silica in experimental groups achieved a lower Ra value compared to the control group. The value of surface roughness (Ra) decreased with increasing hydroxyl content. As a result, TMAOH exhibited the lowest Ra value. Regarding MEA and EDA, although EDA as a catalyst resulted in higher hydroxyl content compared to MEA, EDA’s higher basicity can cause some degree of etching on the silicon wafer surface. Consequently, the Ra value for the MEA group was lower than that for the EDA group.

**Figure 9:**
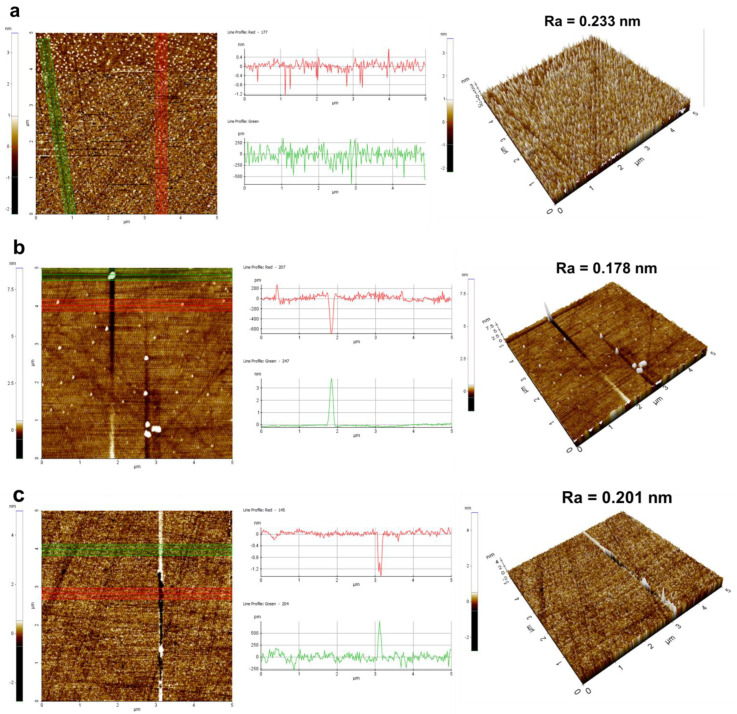

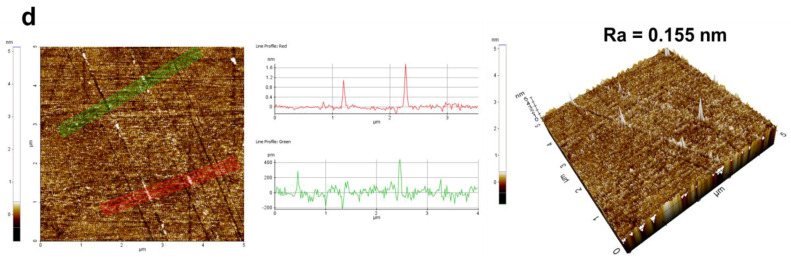
The surface morphology and scratch performance of silicon wafer: (**a**) control group, (**b**) MEA, (**c**) EDA, and (**d**) TMAOH (the red and green lines characterized the degree of particles aggregation and the depth of scratches).

A detailed analysis of the polished silicon wafer surfaces of four sets of samples is provided in Figure 9. In the surface images, highlighted by red and green lines, particle residues and surface scratches are discernible. The accompanying 3D morphology images offered a more intuitive representation of surface quality post-polishing. The control group’s surface morphology revealed numerous particle residues, and the scratch depth was 1.2 nm. Compared to the control group (Figure 9a), MEA-catalyzed silica sol (Figure 9b) exhibited a reduction in particle residues and scratch depth (400 pm), although some particle aggregation was evident. EDA-catalyzed silica sol (Figure 9c) showed a significant decrease in residue particles, yet distinct scratches were noticeable (1.6 nm). In contrast, TMAOH-catalyzed silica sol (Figure 9d) displayed fewer particles and scratches with enhanced surface flatness (200 pm). The aforementioned analysis demonstrated that utilizing organic amine-catalyzed silica sol as a silicon wafer polishing solution can variably reduce particle residues and scratches, with TMAOH proving the most effective catalyst.

#### 3.3.2. Electrochemical Analysis

**Figure 10:**
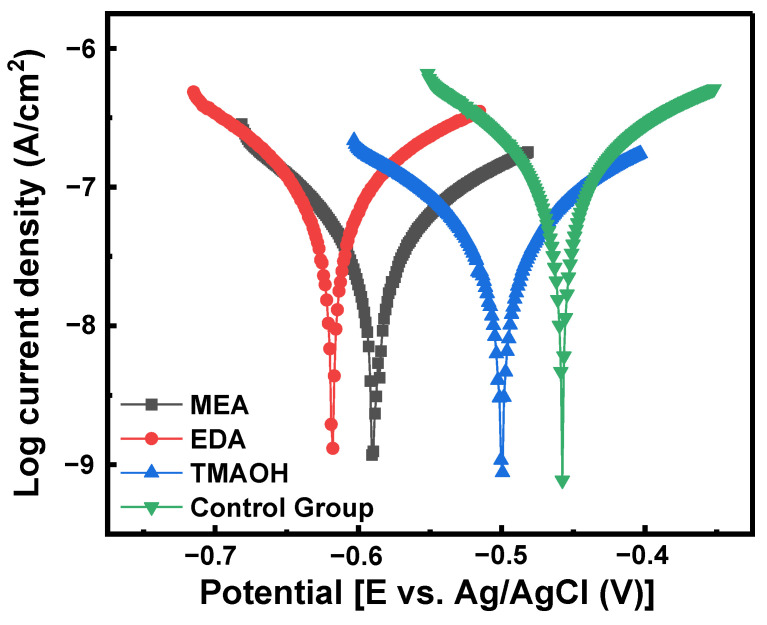
Potentiodynamic polarization curve of different components in polishing slurries at pH 10.50.

The potential polarization diagrams of silicon in slurries from both the control group and the experimental groups are presented in Figure 10. Table 3 illustrates the corrosion potential (Ecorr), corrosion current density (Icorr), and inhibition efficiency (IE%). Ecorr and Icorr were determined using the Tafel method, while IE% was calculated using Equation (9). Compared to the control group, the corrosion current density decreased in the other three experimental groups using MEA-, EDA-, and TMAOH-catalyzed silica sols. Specifically, TMAOH-catalyzed silica sol exhibited the lowest corrosion current density and highest inhibition efficiency, indicating the formation of a passivation film on the silicon wafer surface and reducing the erosive impact of the polishing slurry, thereby improving the surface quality post-polishing. In addition, the protective effect of the TMAOH group was superior to that of the MEA group and EDA group.

(9) $$\mathrm{IE}\%=\frac{{\mathrm{Icorr}}_{0}-\mathrm{Icorr}}{{\mathrm{Icorr}}_{0}}$$

## 4. Conclusions

This study explored the direct synthesis of alkaline silica sol using MEA, EDA, and TMAOH as catalysts for silicon wafer polishing fluids, contrasting it with the traditional method of neutral silica sol with added alkalis. The approach not only made the polishing fluid stable but also enhanced polishing efficiency and improved silicon wafer surface flatness. In addition, TMAOH-catalyzed silica sol exhibited the lowest corrosion current density and highest inhibition efficiency, as evidenced by AFM and electrochemical experiments. This enhancement was attributed to the increased hydroxyl content on silica surfaces, facilitating hydrogen bond formation with amine and water molecules. Additionally, the dehydrogenation of silanol groups created negatively charged sites capable of binding with organic amine cations, thereby enhancing electrostatic repulsion. These findings carry substantial implications for silicon wafer polishing and semiconductor manufacturing processes.

## Figures and Tables

**Table 1:** Si CMP process parameters.

Process Parameters	Value
Polishing time	10 min
Pad/Head rotation speed	40/40 rpm
Wafer rotation speed	30 rpm
Slurry flow rate	155 mL/min
Pressure/Down force	2 psi

**Table 2:** Summary of pore properties of silica particles synthesized using different organic amines.

Parameter	Samples		
	MEA	EDA	TMAOH
S_BET_ ^a^ (m^2^/g)	44.22	10.02	23.34
S_micro_ ^b^ (m^2^/g)	5.42	0.75	13.63
S_micro_/S_BET_ (%)	12.48	7.48	58.39
V_t_ ^c^ (cm^3^/g)	0.29	0.04	0.12
V_micro_ ^d^ (cm^3^/g)	2.7 × 10^−3^	3.18 × 10^−4^	2.9 × 10^−3^
V_micro_/V_t_	0.93	0.79	2.41

^a^ total specific surface area including mesopores and micropores; ^b^ specific surface area of the micropores; ^c^ total pore volume including mesopores and micropores; ^d^ micropore pore volume.

**Table 3:** Corrosion potentials and corrosion current densities for silicon in the polishing slurry at pH 10.50.

Slurry	Components (pH = 10.50)	Ecorr (V)	Icorr (A/cm^2^)	IE%
1	Control Group	−0.4577	3.7246 × 10^−7^	/
2	SiO_2_ + MEA	−0.4999	4.5896 × 10^−8^	87
3	SiO_2_ + EDA	−0.5679	4.1846 × 10^−8^	88
4	SiO_2_ + TMAOH	−0.5892	3.7797 × 10^−8^	89

## Data Availability

The data are contained within the article.
